# Conus medullaris and cauda equina syndrome as complications of non-imaging guided epidural steroid injection: two case reports with comprehensive interdisciplinary work-up

**DOI:** 10.1186/s12883-025-04377-0

**Published:** 2025-08-27

**Authors:** Oliver Gross, Kerstin Schweyer, Benjamin Fritz, Thomas M. Kessler, Martin Schubert, Armin Curt, Carl M. Zipser

**Affiliations:** 1 Spinal Cord Injury Center, Balgrist University Hospital, University of Zürich, Zürich, Switzerland; 2 Department of Neuro-Urology, Balgrist University Hospital, University of Zürich, Zürich, Switzerland; 3 Department of Radiology, Balgrist University Hospital, University of Zürich, Zürich, Switzerland

**Keywords:** Spinal cord injury, Epidural steroid injection, Complication, Neurophysiology, Neuro-urology

## Abstract

**Introduction:**

Epidural steroid injection (ESI) is commonly performed in the outpatient setting for relieving lumbosacral radicular pain, i.e., sciatica. Neurological and neuro-urological adverse events are rare but devastating when occurring. This led to a warning of the U.S. Food and Drug Administration about the use of corticosteroids in the spine. Reviews on causes and numbers of complications are lacking.

**Case presentation:**

Two cases from our tertiary spine center who sustained conus medullaris and cauda equina syndrome following ESI in a secondary care setting not affiliated with our institution. Both patients received ESI for the treatment of chronic lower back pain without sciatica and any reported neurological impairment prior to the injection. In both Case 1 (F, 72yrs) and Case 2 (F, 72yrs), ESI was administered without imaging-guidance and without pre-interventional lumbar MRI. We assessed both patients by thorough neurological examination, comprehensive neuroimaging, neurophysiology, and neuro-urological assessments. Case 1 had cauda equina syndrome, arachnoiditis was diagnosed from lumbar MRI. Case 2 had conus medullaris syndrome probably related to a reported accidental dura puncture. Symptoms of lumbosacral sensory impairment partly recovered, motor symptoms recovered, but neurogenic lower urinary tract and bowel dysfunction persisted. One patient still requires intermittent self-catheterization, while the other patient suffers from bowel dysfunction at 2-yr follow up.

**Discussion:**

Although neurological complications from ESI are rarely reported they can be associated with serious long-term impairments. Comprehensive diagnostic work-up is required to discern potential underlying pathomechanisms and to quantify neural damage load. Neuro-urological diagnostics is required to reveal bladder-bowel dysfunction, install proper management and to prevent secondary complications. We advocate imaging-guidance in ESI to reduce the rate of neurological complications.

## Introduction

Epidural steroid injection (ESI) is a common outpatient procedure for pain relief in patients suffering from lumbosacral radicular pain, i.e., sciatica, due to degenerative lumbar spine disease. Although corticosteroids are commonly used for epidural injections worldwide, concerns remain regarding their safety. In 2014, the U.S. Food and Drug Administration (FDA) issued a warning about the use of corticosteroids in the spine, stating: “*Injection of corticosteroids into the epidural space of the spine may result in rare but serious adverse events*,* including loss of vision*,* stroke*,* paralysis*,* and death.*” [1]. This warning was added to the labels of injectable corticosteroids to highlight these risks. In response, several professional societies raised concerns that the FDA’s warning did not adequately differentiate the risks and benefits associated with various injection routes (transforaminal vs. interlaminar) or between particulate and non-particulate steroid formulations.

Several cases were reported with irreversible spinal cord and radicular nerve damage related to spinal ischemia following ESI probably caused by accidental puncture of the radiculomedullary artery [2–6]. Patients with proof of spinal ischemia as diagnosed from lumbar spinal cord MRI developed neuro-urological problems as well as paraplegia lasting mostly more than one year. Other reports describe paraplegia after lumbar injection in patients with an unrecognized spinal dural arteriovenous fistula [7, 8], unrecognized paraganglioma [9], high grade lumbar stenosis [10], epidural hematoma [11] or lumbar disc herniation [12] suggesting a “mechanical” problem caused by the elevated pressure after injection on the nerve roots. However, there are no rigorous reviews on reported incidence of neurological complications after epidural steroid injection.

Here we present two cases with incomplete acute conus medullaris and cauda equina syndrome that were referred to our tertiary center. Both patients provide written informed consent to the publication, have seen the images and read the article, and were legally entitled to give this consent. These case reports are unique for the comprehensiveness of the interdisciplinary investigations and management, as well as the longish follow-up, including neuroimaging, neurophysiology, and neuro-urology. The case reports are followed by a discussion of pathophysiological and radiological considerations. The case presentations follow the CARE guidelines for case reports [13].

### Case reports

#### Case 1

Prior to the injection the patient (F, 72yrs) had lower back pain, but did not report any neurological impairment (key findings from both cases are summarized in Table 1). From the secondary care medical report, the patient had an uncomplicated ESI at level S2 (side and transforaminal or interlaminar approach not reported) with an unspecified combination of local anesthetic and steroid. There was no written documentation or recordings from imaging provided, i.e., no evidence of ultrasound guidance. Immediately after the injection, the patient had difficulties in walking and incomplete sensorimotor paraplegia. Motor symptoms completely recovered, sensory impairments partly recovered within 24 h. At the emergency presentation at our tertiary care hospital, there was residual numbness in the right S3 dermatome, and bilateral numbness and reduced pin prick sensation in S4/5 dermatomes, voluntary sphincter contraction was weak but preserved. There was loss of sensation for bladder filling and trouble of voiding, as well as bowel dysfunction. The clinical findings were compatible with an incomplete cauda equina lesion. Uroflow showed reduced urine flow during voluntary micturition and ultrasound of the bladder revealed 180 mL of residual urine after spontaneous voiding of 100 mL.

**Table 1:** Key findings in case 1 and 2. EMG: electromyography; ESI: epidural steroid injection; NCS: nerve conduction studies; SSEP: somatosensory evoked potentials

	Case 1	Case 2
Demographics	F, 72 yrs	F, 72 yrs
Intervention	ESI at level S2 (drugs not specified)	ESI at level L5/S1 with bupivacaine 0.5% and betamethasone
Acute symptoms	Incomplete sensorimotor paraplegia, loss of sensation for bladder filling	Thoracic sensory level and loss of sensation for bladder filling, bowel dysfunction
Clinical findings and syndrome	Sensory deficits S3-S5, cauda equina syndrome	Sensory level T10, conus medullaris syndrome
MR- imaging	Initial anterior displacement of the cauda equina nerve roots, at follow-up consistent with arachnoiditis	No abnormalities
Neurophysiology	Abnormal pudendal SSEP and tibial nerve NCS at initial visit	Normal pudendal SEP and tibial nerve NCS, denervation of anal sphincter in needle EMG
Neuro-Urology and bowel function	Acontractile detrusor, post-void residual urine volume 210 mL	Stress urinary incontinence with preserved spontaneous voiding and no post-void residual, acontractile detrusor, interchanging diarrhea- obstipation episodes, stool incontinence
Medical management	Intermittent self-catheterization	Dietary bowel management

**Fig. 1:**
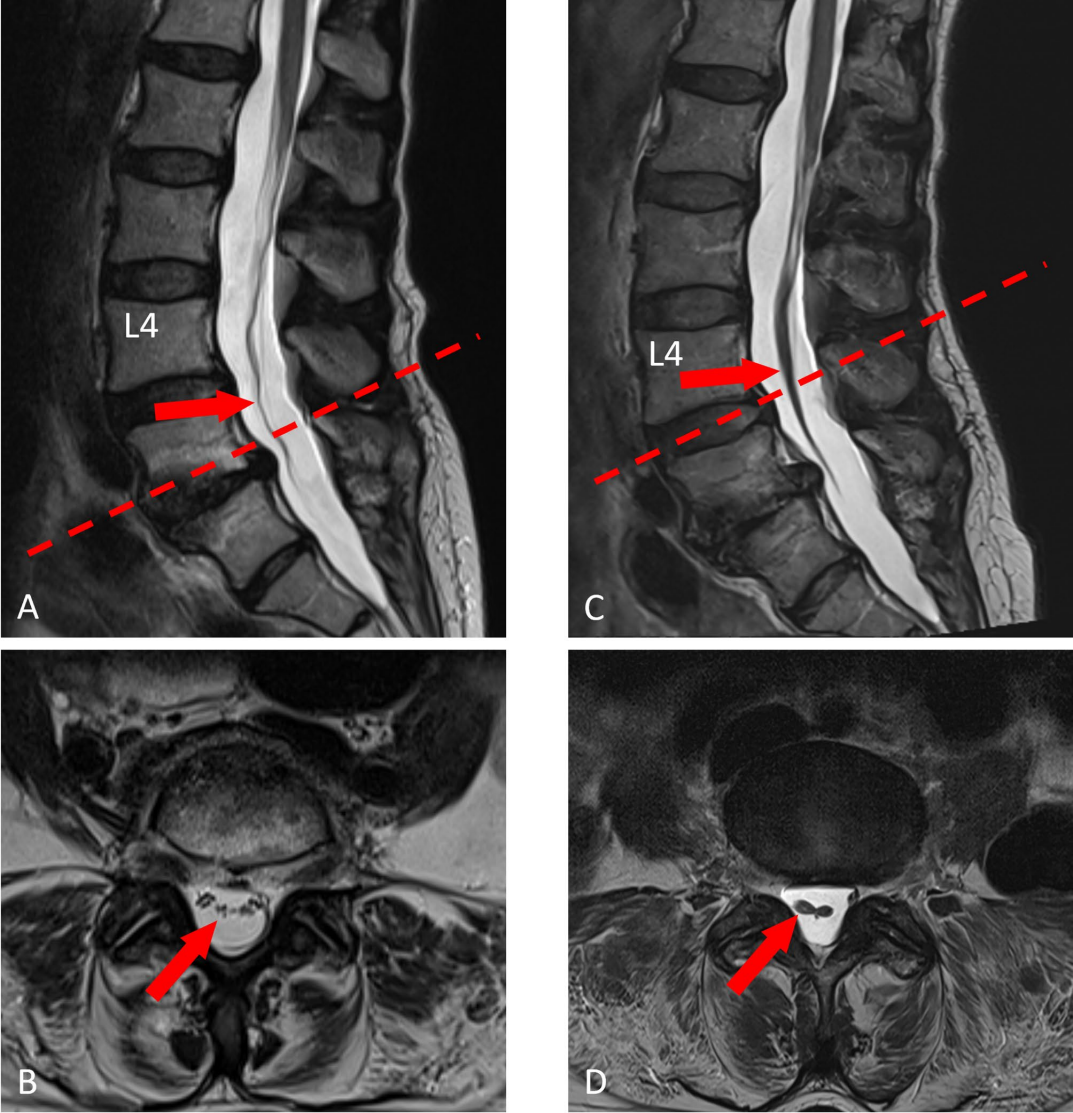
MRI of the lumbar spine of Case 1 one day (**A**, **B**) and two years (**C**, **D**) after the epidural steroid injection with sagittal (**A**, **C**) and transverse (**C**, **D**) T2-weighted sections. The MRI one day after the injection shows an anterior displacement of the cauda nerve roots from the L3 to S1 level (arrows in **A** and **B**), suggesting adhesions of the nerve roots to the dural sac and possibly an early sign of arachnoiditis. The follow-up MRI two years after the injection shows a clumping of the cauda nerve roots to a central cord-like structure (arrows in **C** and **D**) and peripheral adherence of the nerve roots to the dural sac distally (not shown), representing a typical finding after arachnoiditis. Note: the dashed line in **A** describes the orientation of the transverse section B at level L5/S1, the dashed line in **C** describes the orientation of the transverse section **D** at the level L4/5

An emergency lumbar spine MRI was performed the day after infiltration, revealing anterior displacement of the cauda equina nerve roots from the L3 to S1 level (Fig. 1A-B). This may be due to adhesions of the nerve roots to the dural sac, potentially indicating early signs of arachnoiditis. Arachnoiditis refers to a chronic inflammatory condition of the arachnoid mater. Histologically, fibrosis and adhesion formation, thickening of the arachnoid, inflammatory cell infiltration, and vascular proliferations and obliterations often occur. In MRI of the lumbar spine, arachnoiditis is characterized by abnormal clumping or adhesion of the cauda equina nerve roots. The nerve roots may appear as a central conglomeration or may adhere peripherally to the margins of the thecal sac, resulting in an empty or irregularly filled central cerebrospinal fluid space. These findings are best appreciated on axial and sagittal T2-weighted sequences [14, 15]. In this case, a pre-existing condition cannot be excluded, as no prior lumbar MRI was available for comparison. However, given absence of neurological deficits prior to ESI, this is unlikely. No mass or fluid collection was observed between the cauda equina nerve roots and the dural sac, and the cerebrospinal fluid displayed normal signals on T1- and T2-weighted sequences throughout the entire lumbar spine. The MRI long-term follow up at two years revealed a newly definable clumping of the descending caudal fibers to a central intradural cord-like structure from level of the conus to L4, as well as marginal adherence of the caudal fibers to the dural sac from L4 to S1 with an “empty thecal sac sign”. The finding is consistent with a status after arachnoiditis (Fig. 1C-D).

**Fig. 2:**
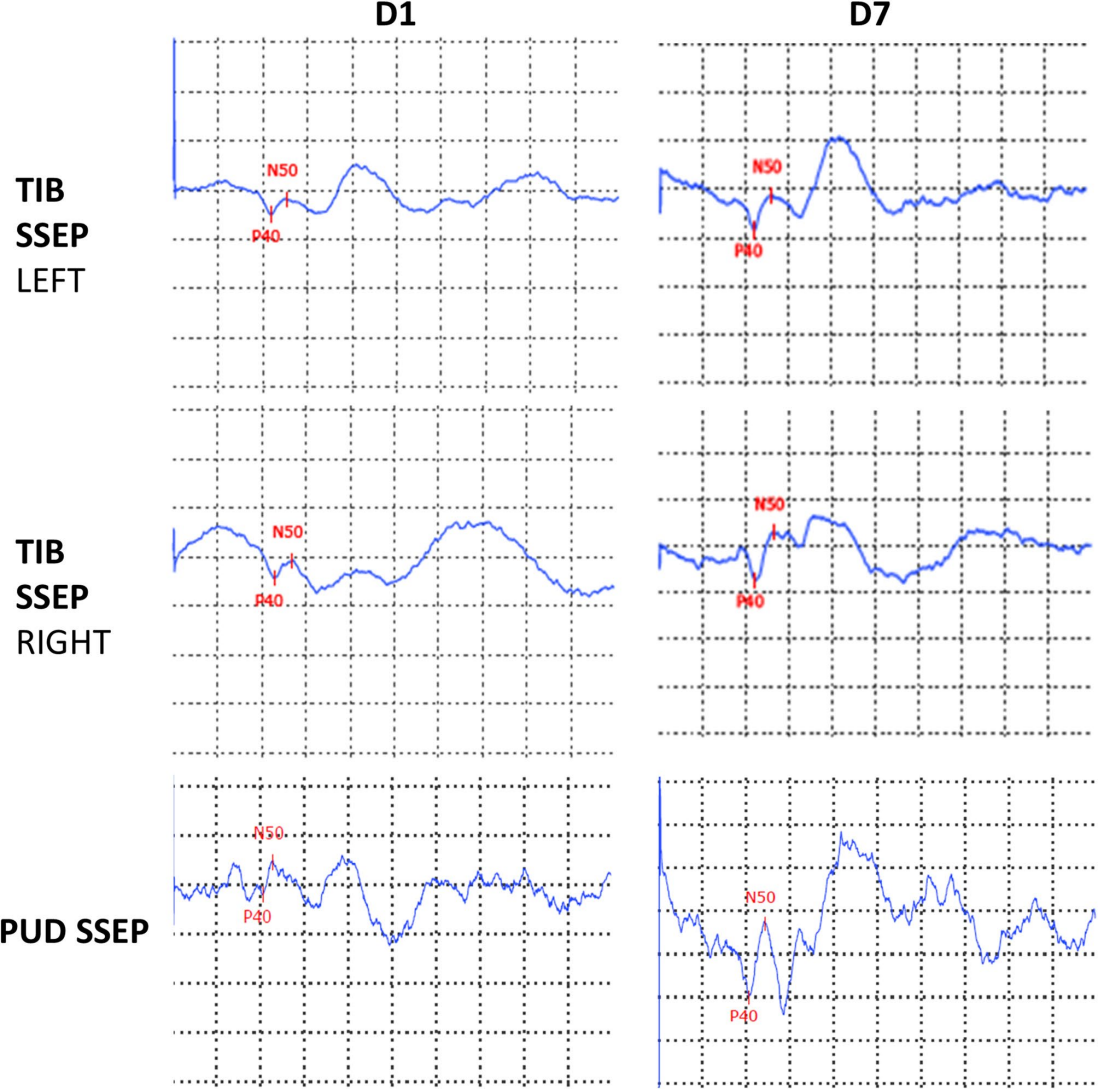
Somatosensory evoked potentials (SSEP) of tibial and pudendal nerve at day 1 (D1) and follow-up (D7) from Case 1. Y-axis: 2 µV/division (for pudendal nerve 0.5µV/division), X-axis: 20 ms/division

Somatosensory evoked potentials (SSEP) of the tibial nerve were normal, whereas pudendal SSEP were of low amplitudes and abnormally configured (Fig. 2A). At the short-term follow-up examination, the pudendal SSEP normalized (in µV: 0.4 at baseline to 0.9 at d7). Additionally, tibial nerve electroneurography (ENG) including F-waves, and motor-evoked potentials (MEP) were performed. Tibial nerve ENG was abnormal with reduced compound muscle action potential amplitude bilaterally (in mV: left/right 1.4/0.7) but normal nerve conduction velocity and F-waves latencies (not shown). Central motor conduction times to the lower extremities were normal. These results suggest lower motoneuron (peripheral) rather than spinal (central) damage.

**Fig. 3:**
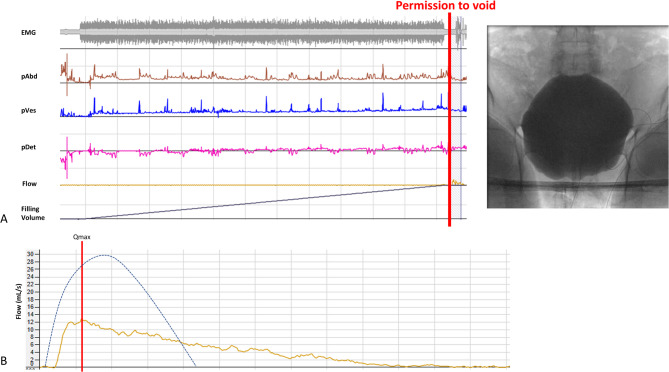
Video-urodynamic findings of Case 1. No detrusor overactivity in filling cystometry and no detrusor contractility after permission to void. The maximum bladder capacity was 490 mL. Fluoroscopy showed a closed bladder neck during the voiding phase, and electromyographically, a non-relaxing pelvic floor was observed (**A**). Free Uroflowmetry of P1 with a prolongated curve and post-void residual of 210 mL. Voided volume: 264 mL, peak urinary flow rate (Qmax): 13.0 mL/s. The blue dotted line shows, for reference, a normal uroflowmetry as would be expected in a healthy individual (**B**)

The neuro-urological management included the placement of an indwelling transurethral catheter, followed by a diagnostic work-up with urethro-cystoscopy and video-urodynamic examination. The urethro-cystoscopy was unremarkable except for mild trabeculation. The video-urodynamic investigation revealed an acontractile detrusor (Fig. 3A). This finding again supported the assumption of lower motor neuron damage. The free uroflowmetry showed a prolonged curve with a post-void residual of 210 mL (Fig. 3B). Therefore, the patient was instructed in intermittent self-catheterization. In the video-urodynamic follow-up investigation after 2.5 years, an acontractile detrusor was still present (not shown). With persistently elevated post-void residual of up to 160 mL, intermittent self-catheterization remained necessary 2.5 years after ESI.

The patient shared following perspective:

*“I will never forget the three hours of horror that followed an intervention intended to reduce my back pain. Immediately after the procedure*,* I tried to get up – only to discover*,* in complete shock*,* that I had absolutely no sensation in my body from the waist down. My greatest fear at that moment was that I might never walk again. I left the hospital with a permanent need for catheterization*,* manual stool evacuation*,* and no sexual function. This condition has made social interactions incredibly difficult. I am unable to control the involuntary passage of air*,* which occurs with noise and without warning. I also experience complete numbness in both the vagina and clitoris – no sexual sensation at all. It is a devastating and deeply isolating experience. How many more patients have to endure years of suffering before procedures like this are properly regulated*,* and these preventable outcomes are no longer allowed to occur?”*

#### Case 2

This patient (F, 72 yrs) suffered from back pain without neurological impairment prior to ESI. From the secondary care medical report, it was retrieved this patient had ESI at level L5/S1 with bupivacaine 0.5% and betamethasone without imaging-guidance (side and transforaminal or interlaminar approach not reported; concentration and volume of drugs not further specified). There was an accidental dura puncture recognized through CSF outflow as reported by the examiner. The needle was slightly retracted and the injection administered.

The patient was presented to our center two days following the injection with a sensory neurological level of T10, with more severe deficits in the S3-5 dermatomes bilaterally, without motor deficits and weak, but intact voluntary sphincter contraction. These findings were suggestive of a conus medullaris lesion. This patient also reported loss of sensation for bladder filling and trouble of voiding, as well as bowel dysfunction. Bowel dysfunction consists of changing episodes of diarrhea and obstipation, as well as stool incontinence.

**Fig. 4:**
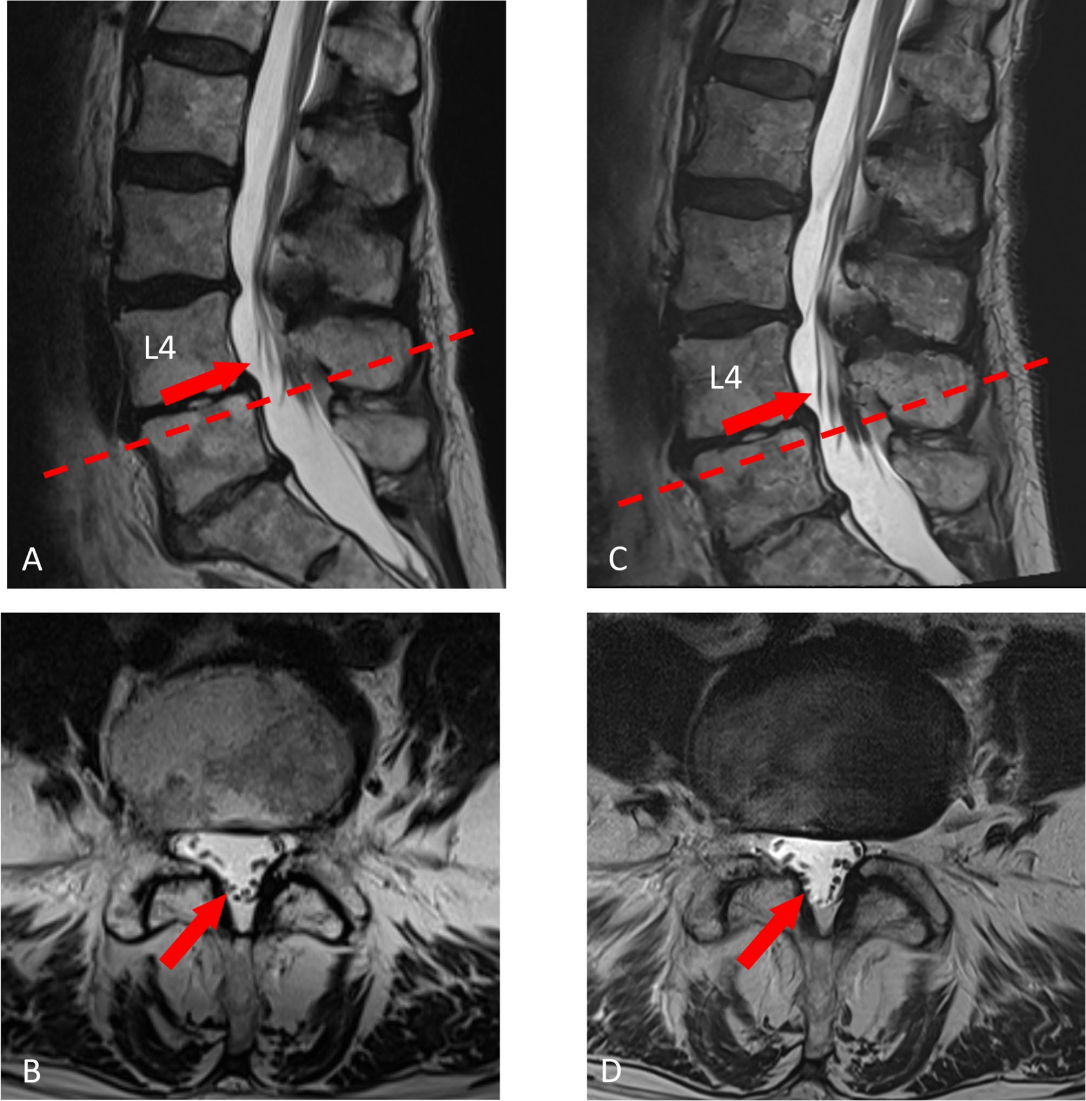
MRI of the lumbar spine of Case 2 one day (**A**, **B**) and two months (**C**, **D**) after the epidural steroid injection with sagittal (**A**, **C**) and transverse (**C**, **D**) T2-weighted sections. Both MRI show moderate degenerations of the lumbar spine but a normal appearance of the conus medullaris and cauda nerve roots and no signs of arachnoiditis (arrows in **A** – **D**). Note: the dashed line in **A** describes the orientation of the transverse section **B** at level L4/5, the dashed line in **C** describes the orientation of the transverse section **D** at the level L4/5

**Fig. 5:**
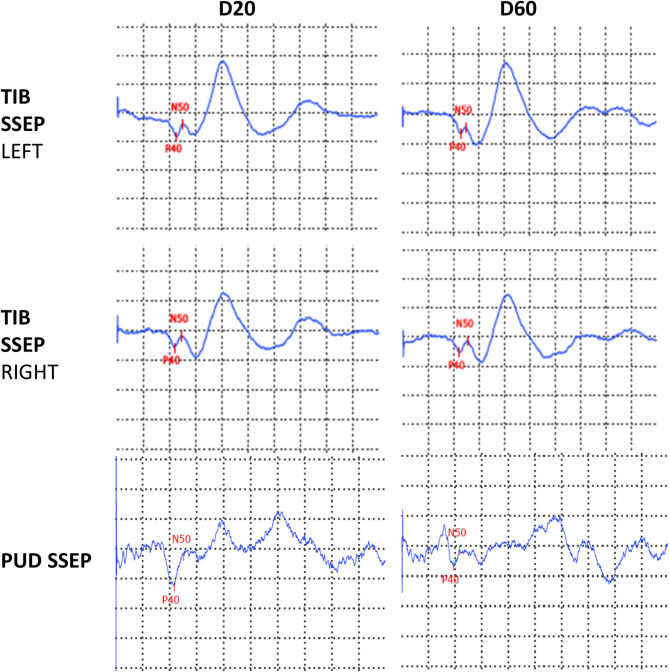
Somatosensory evoked potentials (SSEP) of tibial and pudendal nerve at day 20 (D20) and follow-up (D60) of Case 2. Y-axis: 2 µV/division (for pudendal nerve 0.5µV/division), X-axis: 20 ms/division

Emergency lumbar spine MRI was performed at day 2 following infiltration to rule out conus ischemia and epidural hematoma (Fig. 4A-B). Tibial and pudendal SSEP were normal (Fig. 5). To further quantify the neurological disturbance in this case, anal sphincter needle-electromyography was performed and signs of denervation at d13 post-infiltration were recorded. In the absence of paraparesis and normal neurographies (see below) this would point to a focal spinal damage of Onuf’s nucleus rather than a cauda lesion. In the short-term follow-up examination (3 months post-infiltration) SSEP remained unchanged. Additionally, tibial nerve ENG including F-waves, and MEP were performed. Tibial nerve ENG had normal amplitudes and normal nerve conduction velocity (in m/s: left/right 50/51) and F-waves latencies. A vascular lesion was considered and a 3-month follow-up MRI including diffusion weighted sequences was scheduled. This MRI did not show any changes compared to the acute MRI, in particular no vascular lesion of the conus medullaris (Fig. 4C-D). However, small vessel ischemia as well as inflammation could go undetected on MRI. Lumbar puncture was not done, as the chance of identifying a causative agent was considered low.

**Fig. 6:**
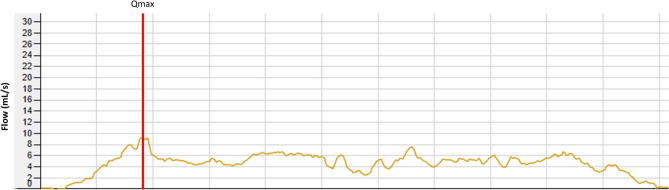
Free Uroflowmetry of Case 2 with a flattened and prolongated curve without post-void residual. Voided volume: 250 mL, peak urinary flow rate (Qmax): 9.2 mL/s

**Fig. 7:**
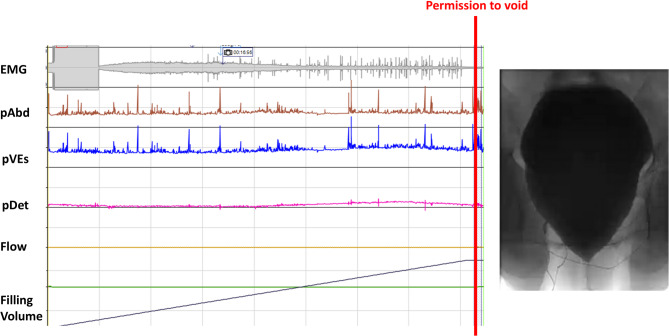
Video-urodynamic findings of Case 2

At the initial neuro-urological evaluation, which took place two days after the ESI, clinical findings included stress urinary incontinence with preserved spontaneous voiding and no post-void residual. In the free uroflowmetry, a flattened flow curve was observed (Fig. 6). The video-urodynamic examination revealed an acontractile detrusor, which did not recover over the course of 2 years (Fig. 7). Bladder emptying was possible likely with abdominal pressure and a weakened urinary stream but without post-void residual. This may only be possible because of reduced sphincter tone due to denervation.

The patient shared following perspective:

*“Before this fatal intervention I just had lower back issues. Now I am dealing with considerable bowel- and bladder- problems which massively impact my quality of life. Sexual activity is no longer possible. My freedom of movement is limited as I cannot leave the house without incontinence pads.”*

## Discussion

### Summary of main findings

The two patients present with incomplete cauda equina (Case 1) and conus medullaris syndrome (Case 2) consisting of saddle anesthesia and neurogenic lower urinary tract and bowel dysfunction following ESI.

### Case-based- pathophysiological considerations and disease course

In Case 1, while initial motor symptoms of the legs improved, sacral sensory deficits and a disabling lower urinary tract and bowel dysfunction persisted over the follow-up period of 2.5 years, suggestive of permanent nerval damage in lower sacral segments. The medical management consisted of intermittent self-catheterization. There were initially pathologic findings in the pudendal SSEP with restitution at short time follow-up, suggestive of a temporary anesthetic effect related to the local anesthetic or neurapraxia. This finding corresponded to the spontaneous recovery from incomplete paraplegia. Paraplegia rehabilitation was not necessary as sensorimotor deficits remitted. Bladder dysfunction was managed in the outpatient setting. There were structural correlates of arachnoiditis in lumbar MRI that evolved over time. This could be related to chronic inflammation of the cauda fibers. Alternatively, we consider drug toxicity related to unrecognized incidental intradural injection of corticosteroids a likely cause.

In Case 2, neuro-imaging was unremarkable with regards to nerval structures. Specifically, there was no evidence for other common complications, such as ischemia (although extent of damage may have been too small to be detected on MRI), cerebrospinal fluid leakage, infection (i.e. epidural abscess or discitis), hemorrhage, or direct nerve damage at the insertion site [16]. As accidental intradural injection might have occurred, given the notion of dural puncture by the examiner, and due to clinical, neurophysiological and urodynamic tests a spinal (conus) lesion most likely occurred. The neurophysiology findings suggested affection of Onuf’s nucleus, a small but essential group of neurons in the ventral horns of the sacral spinal cord, which are potentially sensitive to chemical irritation [17]. The medical management consisted of dietary bowel management.

### Radiological considerations

Current evidence suggests small treatment effects from ESI for reducing leg pain and disability at short term follow-up [18]. Notably, ESI is usually not recommended in patients with lower back pain without sciatica as done in these cases [19]. Given that ESI was recommended in a secondary care setting not affiliated with our hospital, we cannot provide further details on the decision-making process. Very recently, a report of the American Academy of Neurology affirms limited evidence and possible short-term effects of ESI in lumbar radiculopathies and lumbar stenosis [20]. Additionally, a recent systematic review concluded that interventional procedures for spine pain may provide little to no pain relief [21]. As for limitations, this review included several outdated studies, many studies did not have baseline spine imaging which is essential for making the correct diagnosis, it was not clearly distinguished between ESI and other types of infiltrations and lastly, most injections were done without imaging guidance. Considering the risk-benefit ratio, the cases presented here highlight the need to have a proper indication for ESI and imaging guidance. In both patients there were clinically no contraindications for epidural infiltration. However, there were no baseline MRI performed before the intervention, which is strongly recommended to confirm the diagnosis and to rule out low lying conus or non-degenerative spine pathologies. Contraindications for ESI can be categorized as absolute or relative. Absolute contraindications include uncorrected coagulopathy, systemic infection, infectious spondylodiscitis, active infection at the injection site, acute spinal cord compression, myelopathy, or cauda equina syndrome. Relative contraindications include, but are not limited to, ongoing anticoagulant therapy, hypersensitivity or allergy to the administered agents, and pregnancy [22]. With the available information, drugs were standard for epidural injection and were administered by an experienced practitioner. In both cases, the injection was done without image-guidance and both patients did not have a pre-interventional lumbar MRI. Various medical societies and expert groups have issued safety guidelines and practice parameters for ESI. While there are some differences among these recommendations, most agree that ESIs should be performed under image guidance, such as fluoroscopy, computed tomography (CT), or ultrasound [23]. Image guidance ensures proper documentation of the procedure, helps achieve correct needle placement, and prevents intrathecal or intravascular injections. Without image guidance, the risk of improper injection increases, with studies showing misplacement during blind ESI in 15–25% even in experienced hands [24, 25]. In contrast, during CT-guided injections, correct placement is reliable, the risk of neurological complications is low, and paraplegia is very rarely reported [26, 27]. Some experts recommended the use of non-particulate steroids, such as dexamethasone, as the preferred option for ESIs over particulate steroids [6, 28, 29]. This preference aims to minimize the risks of particle aggregation, embolization, small vessel occlusion, and the subsequent risk of neural ischemic injury [30]. Most expert groups agree that ESIs should only be performed by highly trained and skilled physicians who possess a deep understanding of all aspects of epidural pain management. This includes thorough knowledge of spinal anatomy, pathology, pathophysiology, the risks and benefits of epidural steroids, as well as the appropriate indications and contraindications for administering ESIs [22].

### General pathophysiological considerations

Several pathomechanisms leading to nerve damage after epidural steroid injection were described previously. In those cases, the transforaminal injection route and the use of particulate steroids were suggested as having a higher risk of spinal ischemia [29, 31]. Furthermore, the impact of different injection volumes was discussed and smaller injection volumes especially in elderly patients were recommended [10]. In other cases, direct neurotoxicity of the injection components was proposed [3, 12, 32] and mainly attributed to the local anesthetic in the injection. In the majority of the reported cases, bupivacaine in a concentration of 0.25–0.75% was used. In fewer cases, lidocaine in concentrations from 1 to 5% was injected. One case describes a young patient with over 16 months persistent lower urinary tract and bowel difficulties after an accidental injection of lidocaine 2% in the subarachnoid space as part of a planned epidural anesthesia [33]. Other authors described paraplegia and lower urinary tract symptoms with electrophysiological signs of acute lumbosacral polyradiculopathy persisting for more than one year after an epidural injection of 0.25% bupivacaine and triamcinolone [10]. Concerning the type of steroid used for injections, most reported cases used different concentrations of triamcinolone. Triamcinolone was found to contain larger particles when compared to betamethasone and therefore was discussed to have a higher risk of vascular complications [34]. A cumulation of spinal ischemia after epidural steroid injection in France was attributed to the commonly used prednisolone acetate which tends to aggregate more easily leading to a higher risk of embolization in the spinal arteria [2]. Direct neurotoxicity of the steroid and its vehicles polyethylene glycol and the preservative benzyl alcohol were discussed as well. Animal models did not provide proof of direct nerve damage after epidural steroid injection but reported nerve injury after intra-fascicular injection in a rat model [34]. Elder patients and those with previous back operations were affected more commonly in the reported case than younger healthy patients [

### Strengths and limitations

This case report provides novel information on the diagnostic workup of complications from ESI. To our knowledge, none of the previously published reports had neurophysiology and neuro-urology work-up or follow-up MRI. Therefore, our cases are of high value for spinal cord-caretakers as well as to interventionalists. A deeper analysis of the pathomechanism was not possible owed to the limited information on the drugs administered (i.e., substance, dosage, volume, batch of medication) and the administration technique (i.e., limited certainty if transforaminal or laminar approach was followed) related to the intervention being done in a non-affiliated secondary care setting. Given the acute onset of symptoms, we omitted lumbar puncture in both patients. In particular in Case 1, CSF analysis could have helped to determine the cause. Our findings suggest high utility of follow-up MRI. The best timing for the MRI cannot be recommended with certainty. Retrospectively, we would consider repeating the lumbar MRI within three weeks to detect arachnoiditis.

## Conclusions

This interdisciplinary case report presents devastating persistent complications of a seemingly routine lumbar ESI. Some cases are preventable with respective precautions, especially imaging-guidance. If complications occur, a thorough spine-neurological examination is necessary, including spine neuroimaging with follow-up. Neuro-urological management is necessary to prevent further complications from lower urinary tract dysfunction and to improve quality of life.

### Key points

- Incomplete lumbosacral spinal cord injury can occur as a rare complication of epidural steroid injection and is associated with loss of quality of life.
- While there are some differences among radiological recommendations, most agree that epidural steroid injection should be performed under image guidance.
- Thorough neurological examination is key to determine the level of lesion in the lumbosacral cord, i.e. cauda equina-, conus- or epi-conus lesion.
- Interdisciplinary neurological, neuro-physiological, neuro-urological and neuro-radiological work-up is essential to confirm the diagnosis.
- Neuro-urological and bowel management is highly important to avoid secondary complications from lower urinary tract dysfunction and to improve quality of life.

## Data Availability

Data can be shared upon reasonable request.

## Abbreviations

CSF: Cerebrospinal fluid
ENG: Electroneurography
ESI: Epidural steroid injection
MEP: Motor evoked potentials
SCI: Spinal cord injury
SSEP: Somatosensory evoked potentials
VUDI: Video-urodynamic investigation
